# Transcriptome Analysis Reveals Common and Distinct Mechanisms for Sheepgrass (*Leymus chinensis*) Responses to Defoliation Compared to Mechanical Wounding

**DOI:** 10.1371/journal.pone.0089495

**Published:** 2014-02-21

**Authors:** Shuangyan Chen, Yueyue Cai, Lexin Zhang, Xueqing Yan, Liqin Cheng, Dongmei Qi, Qingyuan Zhou, Xiaoxia Li, Gongshe Liu

**Affiliations:** 1 Key Laboratory of Plant Resources, Institute of Botany, the Chinese Academy of Sciences, Beijing, P. R. China; 2 Graduate Schoo1 of the Chinese Academy of Sciences, Beijing, P. R. China; 3 Entry-Exit Inspection and Quarantine Bureau, Heze, Shandong, P.R. China; Nazarbayev University, Kazakhstan

## Abstract

**Background:**

Herbivore grazing is a multiple-component process that includes wounding, defoliation, and saliva deposition. Despite the extensive published research on mechanical wounding and defoliation, no analysis to identify the genes that specify defoliation and mechanical wounding has been performed. Moreover, the influence of the expression of these genes on plant regrowth after defoliation remains poorly understood.

**Results:**

Seven cDNA libraries for RNA samples collected from stubble tissues that had been mechanically wounded or defoliated at 2, 6 and 24 h along with the control were sequenced using the Illumina/Solexa platform. A comparative transcriptomic analysis of the sequencing data was conducted. In total, 1,836 and 3,238 genes were detected with significant differential expression levels after wounding and defoliation, respectively, during one day. GO, KOG and pathway-based enrichment analyses were performed to determine and further understand the biological functions of those differentially expressed genes (DEGs). The results demonstrated that both wounding and defoliation activated the systemic synthesis of jasmonate (JA). However, defoliation specifically reduced the expression levels of ribosomal protein genes, cell division or cell expansion-related genes, and lignin biosynthesis genes and may have negatively affected plant growth. Further analysis revealed that the regrowth of elongating leaves was significantly retarded after defoliation at 6 h through the following 7 days of measurement, suggesting that the gene expression pattern and phenotype are consistent. Fifteen genes were selected, and their expression levels were confirmed by quantitative RT-PCR (qRT-PCR). Thirteen of them exhibited expression patterns consistent with the digital gene expression (DGE) data.

**Conclusions:**

These sequencing datasets allowed us to elucidate the common and distinct mechanisms of plant responses to defoliation and wounding. Additionally, the distinct DEGs represent a valuable resource for novel gene discovery that may improve plant resistance to defoliation from various processes.

## Introduction

Grasslands are a major part of the global ecosystem, covering 37% of the Earth's terrestrial area [1]. Herbivory is a fundamental driver of grassland plant community composition. Nearly all grasslands worldwide have been grazed by populations of wild and domestic herbivores, such as cattle [2], horses [3], sheep [4], deer [5], rabbits [6], and kangaroos [7]. Herbivore grazing is a multiple-component process that includes wounding, defoliation, and saliva deposition [8].

Wounding responses have been demonstrated in species throughout the plant kingdom, and previous research has focused on identifying systemic wound signals and the mechanisms by which they are generated, transported, and perceived [9]. Leaf wounding is a well-characterized stimulus for the rapid activation of jasmonate (JA) synthesis through distinct autonomous and non-autonomous cellular pathways [9]–[12].

Defoliation not only induces serious wounding but also removes a large fraction of photosynthetically active leaves from grass plants. Therefore, plant photosynthesis is reduced; carbohydrate reserves stored in the plant are mobilized; and the transcript levels of photosynthesis-related and carbohydrate metabolism-related genes, such as genes encoding the small subunits of Rubisco, Rubisco activase, light harvesting chlorophyll a/b binding proteins, fructan exohydrolase (FEH), sucrose transporter (SUT), invertase, β-amylase, and starch synthase, are changed [13]–[18].

We previously identified 466 genes that responded to simulated grazing using the Affymetrix Rice GeneChips. These genes encode proteins that participate in signal transduction, miRNA regulation, cell wall modification, carbohydrate metabolism, hormone synthesis, and molecular transport [8]. However, it is unclear whether the responses were due to wounding, defoliation, or other factors.

Currently, the genes that specify defoliation and mechanical wounding remain unknown, and little is understood about how expression of these genes influences plant regrowth after defoliation. In this study, the transcriptomes of sheepgrass stubble tissues collected from defoliated and mechanically wounded plants were compared using the Solexa sequencing system. By comparing the differentially expressed gene (DEG) responses to defoliation and wounding, the common and distinct pathways and genes between defoliation and wounding were identified. These sequencing datasets allowed us to elucidate the common and distinct mechanisms of the plant response to defoliation and wounding, and the distinct DEGs represent a valuable resource for novel gene discovery that may improve plant tolerance toward defoliation caused by herbivores, insects, pathogens, or mechanical stress.

## Materials and Methods

### Plant Materials, Growing Conditions, and Treatments

The sheepgrass variety Zhongke 3 was grown in a soil mix of peat moss and vermiculite (2∶1, v/v) in a greenhouse at 23°C with a photoperiod of 16 h light/8 h dark. Wounding and defoliation treatments were performed on eight-week-old sheepgrass seedlings. To induce defoliation, approximately two-thirds of the aboveground biomass was removed, and to induce wounding, tweezers were used to mechanically wound a site at the same position as the defoliation site. The remaining one-third of the aboveground biomass was collected 2, 6, and 24 h after defoliation, and the corresponding parts were collected for control seedlings and wounding at 2, 6, and 24 h. Each sample included 3 replicate pots and 21 seedlings. The samples were collected and immediately frozen in liquid nitrogen and stored at −80°C.

### RNA Extraction and Solexa/Illumina Sequencing

Total RNA was extracted from individual control, wounded and defoliated plants at 2, 6, and 24 h using TRIzol reagent (Invitrogen, Carlsbad, CA, USA). After precipitation, the RNA was purified using Qiagen’s RNeasy Kit with on-column DNase I digestion according to the manufacturer’s instructions. RNA concentrations were measured using the Bioanalyzer 2100 (Agilent Technologies, Inc., Waldbronn, Germany), and the RNA integrity was analyzed on a 1.0% (w/v) agarose gel. Approximately 5 to 8 µg total RNA was used to construct each RNA-seq library. Poly (A) mRNAs were isolated from the total RNA using oligo (dT) magnetic beads (Illumina, San Diego, CA). RNA fragmentation, cDNA synthesis, and PCR amplification were performed according to the Illumina RNA-seq protocol (Cat # RS-100-0801). Seven cDNA libraries, which were isolated from mechanically wounded and defoliated material at 2, 6, and 24 h and the control, were sequenced using the Illumina HiSeq 2000 System at the Chinese National Human Genome Center (Shanghai, China). The sequence reads generated in this study have been deposited in the NCBI sequence read archive (SRA065691).

### Analysis and Screening of Differentially Expressed Genes (DEGs)

The reads for each sample were counted and mapped back to previously generated sheepgrass reference genes [19] using the software tool Bowtie [20]. The read number of each gene was transformed into RPKM (Reads Per Kilobases per Million reads) [21], and the differently expressed genes were identified by a DEGseq package using the MARS method (MA-plot-based method with Random Sampling model) [22]. The p-value was obtained from the differential gene expression test. FDR (False Discovery Rate) manipulation was used to determine the p-value threshold in multiple tests and analyses. Both FDR ≤0.001 and the absolute value of | the log2Ratio ≥1 were used as thresholds to identify the significant DEGs [23].

### Gene Ontology, EuKaryotic Orthologous Groups and Pathway Enrichment Analysis

Gene Ontology (GO) is an international standardized functional gene classification system that describes the properties of genes and their products in any organism using three ontologies: cellular component, molecular function, and biological process. GO enrichment analysis was performed using agriGO (http://bioinfo.cau.edu.cn/agriGO/, [24]). EuKaryotic Orthologous Groups (KOG) enrichment analysis was conducted through hypergeometric distribution testing using the Phyper function in the R software package (http://www.rproject.org/). The Bonferroni correction was used to adjust the p-values. The significantly enriched functional clusters were selected based on a corrected q-value less than 0.05. Kyoto Encyclopedia of Genes and Genomes (KEGG) pathway enrichment analysis was conducted using KOBAS 2.0 (http://kobas.cbi.pku.edu.cn/, [25]).

### Real-time Quantitative RT-PCR (qRT-PCR) Analysis

Real-time quantitative RT-PCR (qRT-PCR) analysis was used to verify the DGE results. The RNA samples used for the qRT-PCR assays were the same as in the DGE experiments. Gene-specific primers were designed according to the reference unigene sequences using Primer Premier 6.0. Fifteen genes were selected from the DEGs under defoliation at 2 h for quantitative RT-PCR assays, and these genes were involved in JA biosynthesis, the gibberellin signaling pathway, cell wall biogenesis/degradation, and starch and sucrose metabolism (Table S1). qRT-PCR was performed according to the manufacturer’s specifications (TaKaRa SYBR Premix Ex Taq II Kit, Dalian, China). The following SYBR Green PCR cycling conditions were used: denaturation at 95°C for 10 s, followed by 40 cycles of 94°C for 5 s and 60°C for 20 s. The PCR experiments were performed using an iQ 5 Multicolor Real-time PCR Detection System (Bio-Rad, USA). The results were normalized to the sheepgrass actin gene, and the relative gene expression levels were calculated using 2^−ΔΔCT^.

## Results

### Wounding and Defoliation Exhibit Distinct DEGs

To identify the DEGs involved in wounding and defoliation, we sequenced seven cDNA libraries and generated 192 million clean reads ranged from 4,811,093 to 6,411,192 per library after filtering the low-quality and adapter sequences. These clean reads were mapped to the sheepgrass reference genes as described in the Materials and methods. In total, 36,413, 35,925, 35,113, 35,929, 34,534, 36,828, and 36,956 unigenes were detected for sequenced libraries 1–7, respectively (Table S2). The reference unigenes mapped by all clean reads were enhanced as the sequencing amount (total read number) increased. However, when the sequencing counts reached 1.5 million reads or higher, the number of detected genes became saturated (Figure S1), indicating that the sequencing depth was sufficient to cover the transcriptome.

**Figure 1:**
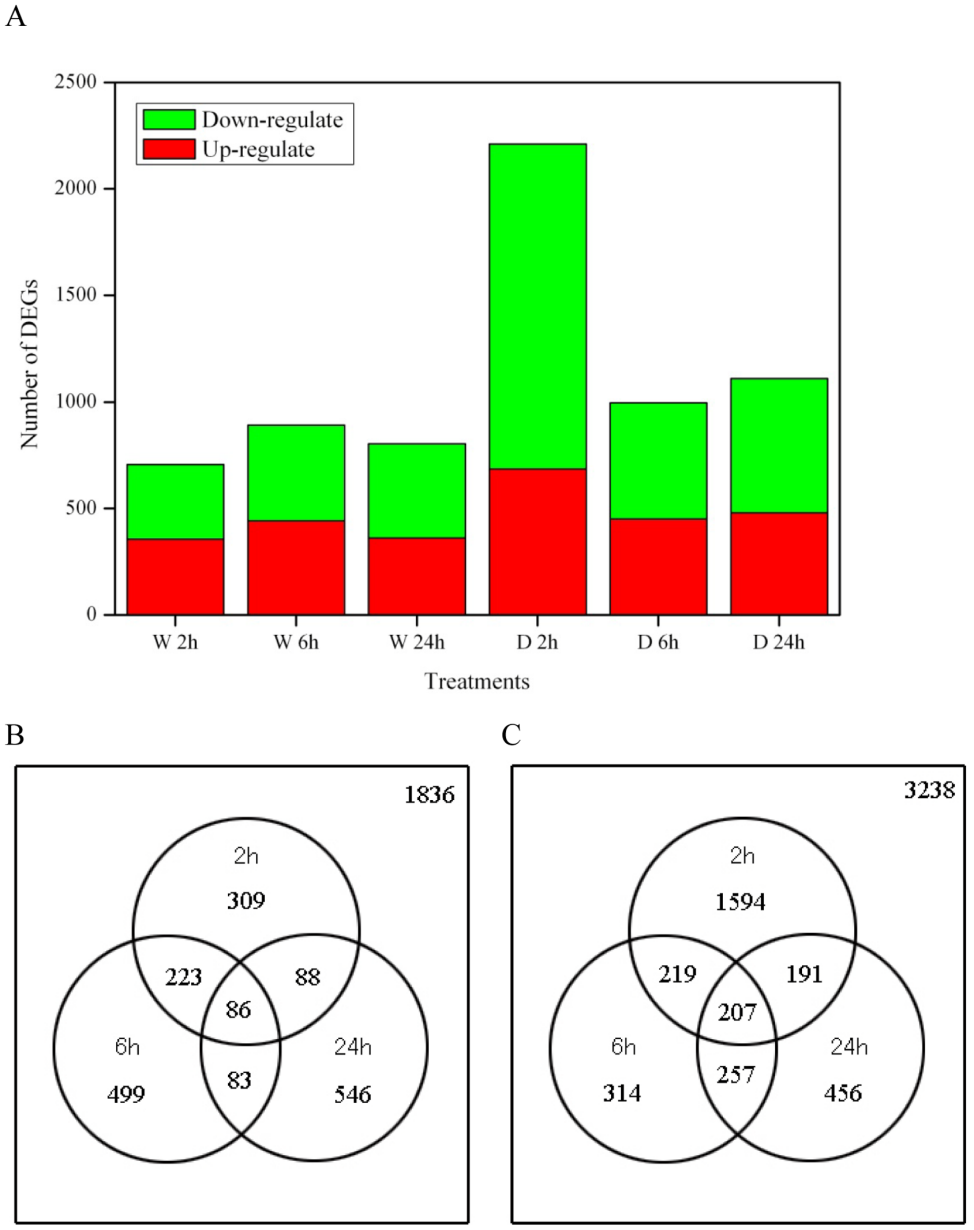
Summary of differentially expressed genes. The categories of DEGs expressed at one time point and at overlapping time points for wounding and defoliation at 2, 6, and 24(A) A summary of the up- and down-regulated DEG numbers. (B) and (C) DEGs expressed at one time point and overlapping time points for wounding and defoliation, respectively.

By comparing the treated and control Solexa libraries, a great number of differentially expressed transcripts were identified. To study the subset of genes associated with wounding and defoliation, we analyzed the most differentially regulated genes with a |log2Ratio|≥1 and a FDR ≤0.001. There were 706 (356 up-regulated and 350 down-regulated), 891 (442 up-regulated and 449 down-regulated), and 803 (362 up-regulated and 441 down-regulated) genes that responded to wounding at 2, 6, and 24 h, respectively. For defoliation, we identified 2,211 (685 up-regulated and 1526 down-regulated), 997 (451 up-regulated and 546 down-regulated), and 1,111 (480 up-regulated and 631 down-regulated) genes that were significantly expressed at 2, 6, and 24 h, respectively (Figure 1A). These significantly differentially wounding- and defoliation-responsive genes were further divided into genes expressed at one time point and those expressed at overlapping time points. For example, 1,836 and 3,238 genes were differentially expressed between the wounding and defoliation treatments, respectively, during one day (Figure 1B, C), and 954 genes were uniquely expressed under only defoliation. These DEGs are listed in Table S3 and Table S4, respectively.

### GO Functional Analysis of DEGs

**Figure 2:**
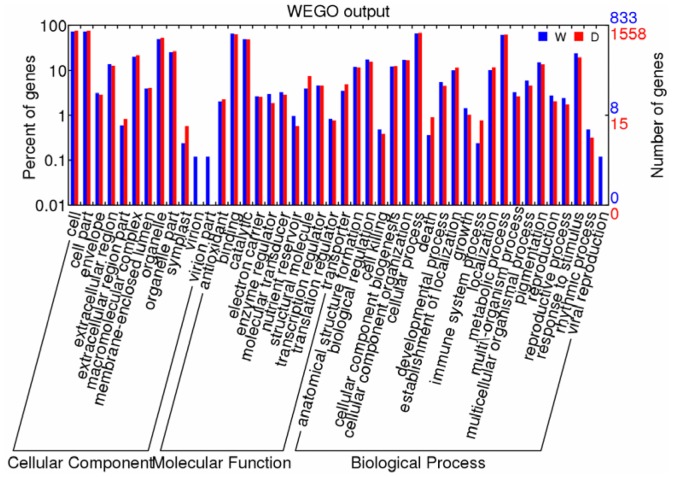
Histogram of functional Gene Ontology analysis of DEGs. The frequency of GO terms was analyzed using the GO Slim Assignment. The x- and y-axes represent the names of clusters and the ratio of each cluster, respectively. W, mechanical wounding; D, defoliation.

A total of 835 DEGs that responded to wounding and 1,560 DEGs that responded to defoliation were divided into 44 GO categories (Figure 2). In the biological process category, cellular processes (wounding 66.6%; defoliation 68.5%) and metabolic processes (wounding 61.5%; defoliation 62.5%) were the most dominant groups, followed by stimulus responses (wounding 23.9%; defoliation 19.4%) and cellular component organization (both approximately 17%). Regarding the molecular function category, 65.9% of the wounding unigenes and 63.5% of the defoliation unigenes were assigned to binding, followed by catalytic activity (both approximately 49%), transcription regulatory activity (4.6% for both), and structural molecule activity (wounding 4.0%; defoliation 7.5%). In the cellular component category, cell and cell part genes (wounding 72.1% and defoliation 75.5% for both) dominated, followed by organelle (wounding 49.9%; defoliation 52.4%) and organelle part genes (wounding 25.5%; defoliation 26.4%).

To reveal significantly enriched GO terms in DEGs compared to the reference transcriptome, GO enrichment analysis of the functional significance was performed. The GO terms with p≤0.05 were considered significantly enriched among the DEGs. This analysis allowed us to determine the major molecular functions, biological processes, and cellular components that were represented among the DEGs (Figure S2). For the molecular functions, the significantly enriched GO terms of the DEGs for wounding and defoliation included DNA binding, serine-type endopeptidase inhibitor activity, oxidoreductase activity, and structural ribosome constituents specific to defoliation. For the enriched biological processes, there were 53 GO terms of DEGs for wounding and 40 GO terms of DEGs for defoliation, which represented cellular component organization and cellular component biogenesis (e.g., chromatin assembly, protein-DNA complex assembly, and nucleosome assembly), metabolic processes (fatty acid and oxylipin biosynthesis), lipid localization, and responses to stimuli (e.g. response to water and defense response to insects). For the cellular components, significantly enriched GO terms of DEGs for wounding and defoliation included chromatin, nucleosome, nucleus, extracellular region, and ribosome, and cell wall was specific to defoliation.

### KOG and KEGG Pathway Enrichment Analysis of DEGs

A total of 832 wounding-responsive DEGs and 1,605 defoliation-responsive DEGs were classified functionally into 24 clusters of KOG. KOG enrichment analyses were performed by hypergeometric distribution testing, and the Bonferroni correction was applied. Significantly enriched functional clusters were selected based on a q-value ≤0.05. The results demonstrated that genes associated with chromatin structure and dynamics, energy production and conversion, and amino acid transport and metabolism were over-represented in the KOG classification of wounding (Table 1), and chromatin structure and dynamics, carbohydrate transport and metabolism, translation, ribosomal structure and biogenesis, and the cytoskeleton were over-represented in the KOG classification of defoliation (Table 2).

**Table 1:** KOG enrichment analysis of the wounding-responsive DEGs compared with the reference transcriptome.

KOG functional cluster	Transcriptome frequency of use	DEGs frequency of use	*p*-value	*q*-value
Chromatin structure and dynamics	636 of 32382 (1.96%)	85 of 832 (10.22%)	7.16E-37	1.79E-35
Energy production and conversion	1062 of 32382 (3.28%)	46 of 832 (5.53%)	0.0002	0.003
Amino acid transport and metabolism	1385 of 32382 (4.28%)	54 of 832 (6.49%)	0.001	0.0085
Carbohydrate transport and metabolism	1897 of 32382 (5.86%)	62 of 832 (7.45%)	0.02	0.14
Defense mechanisms	333 of 32382 (1.03%)	12 of 832 (1.44%)	0.09	0.43
Secondary metabolites biosynthesis, transport and catabolism	1511 of 32382 (4.67%)	46 of 832 (5.53%)	0.1	0.43
Nucleotide transport and metabolism	398 of 32382 (1.23%)	13 of 832 (1.56%)	0.15	0.53
Cell wall/membrane/envelope biogenesis	424 of 32382 (1.31%)	13 of 832 (1.56%)	0.21	0.64
Inorganic ion transport and metabolism	942 of 32382 (2.91%)	27 of 832 (3.25%)	0.24	0.67
Extracellular structures	15 of 32382 (0.05%)	0	0.32	0.77
Cell motility	16 of 32382 (0.05%)	0	0.34	0.77
Transcription	1520 of 32382 (4.69%)	40 of 832 (4.81%)	0.39	0.83
Cytoskeleton	1020 of 32382 (3.15%)	26 of 832 (3.13%)	0.46	0.88
Lipid transport and metabolism	1228 of 32382 (3.79%)	31 of 832 (3.73%)	0.49	0.88
Translation, ribosomal structure and biogenesis	1819 of 32382 (5.62%)	44 of 832 (5.29%)	0.63	1
Posttranslational modification, protein turnover, chaperones	3144 of 32382 (9.71%)	77 of 832 (9.25%)	0.65	1
Coenzyme transport and metabolism	322 of 32382 (0.99%)	4 of 832 (0.48%)	0.92	1
Nuclear structure	142 of 32382 (0.44%)	0	0.98	1
Replication, recombination and repair	821 of 32382 (2.54%)	12 of 832 (1.44%)	0.98	1
Intracellular trafficking, secretion, and vesicular transport	1589 of 32382 (4.91%)	28 of 832 (3.37%)	0.98	1
Signal transduction mechanisms	5114 of 32382 (15.79%)	107 of 832 (12.86%)	1	1
Cell cycle control, cell division, chromosome partitioning	683 of 32382 (2.11%)	8 of 832 (0.96%)	1	1
General function prediction only	3292 of 32382 (10.17%)	63 of 832 (7.57%)	1	1
Function unknown	1685 of 32382 (5.20%)	21 of 832 (2.52%)	1	1
RNA processing and modification	1384 of 32382 (4.27%)	13 of 832 (1.56%)	1	1

**Table 2:** KOG enrichment analysis of the defoliation-responsive DEGs compared with the reference transcriptome.

KOG functional cluster	Transcriptome frequency of use	DEGs frequency of use	*p*-value	*q*-value
Chromatin structure and dynamics	636 of 32382 (1.96%)	144 of 1605 (8.97%)	9.95E-56	2.49E-54
Carbohydrate transport and metabolism	1897 of 32382 (5.86%)	156 of 1605 (9.72%)	1.53E-10	1.92E-09
Cytoskeleton	1020 of 32382 (3.15%)	73 of 1605 (4.55%)	0.0007	0.0045
Translation, ribosomal structure and biogenesis	1819 of 32382 (5.62%)	126 of 1605 (7.85%)	5.97E-05	0.0005
Inorganic ion transport and metabolism	942 of 32382 (2.91%)	59 of 1605 (3.68%)	0.03	0.14
Secondary metabolites biosynthesis, transport and catabolism	1511 of 32382 (4.67%)	87 of 1605 (5.42%)	0.06	0.27
Cell wall/membrane/envelope biogenesis	424 of 32382 (1.31%)	27 of 1605 (1.68%)	0.07	0.27
Defense mechanisms	333 of 32382 (1.03%)	21 of 1605 (1.31%)	0.11	0.29
Nucleotide transport and metabolism	398 of 32382 (1.23%)	25 of 1605 (1.56%)	0.09	0.29
Extracellular structures	15 of 32382 (0.05%)	1 of 1605 (0.06%)	0.17	0.42
Energy production and conversion	1062 of 32382 (3.28%)	58 of 1605 (3.61%)	0.19	0.45
Transcription	1520 of 32382 (4.69%)	77 of 1605 (4.80%)	0.39	0.78
Nuclear structure	142 of 32382 (0.44%)	7 of 1605 (0.44%)	0.41	0.78
Lipid transport and metabolism	1228 of 32382 (3.79%)	61 of 1605 (3.80%)	0.46	0.82
Cell motility	16 of 32382 (0.05%)	0	0.56	0.93
Posttranslational modification, protein turnover, chaperones	3144 of 32382 (9.71%)	152 of 1605 (9.47%)	0.61	0.95
Amino acid transport and metabolism	1385 of 32382 (4.28%)	56 of 1605 (3.49%)	0.94	1
Coenzyme transport and metabolism	322 of 32382 (0.99%)	12 of 1605 (0.75%)	0.81	1
Replication, recombination and repair	821 of 32382 (2.54%)	23 of 1605 (1.43%)	1	1
Intracellular trafficking, secretion, and vesicular transport	1589 of 32382 (4.91%)	50 of 1605 (3.11%)	1	1
Signal transduction mechanisms	5114 of 32382 (15.79%)	159 of 1605 (9.91%)	1	1
Cell cycle control, cell division, chromosome partitioning	683 of 32382 (2.11%)	18 of 1605 (1.12%)	1	1
General function prediction only	3292 of 32382 (10.17%)	120 of 1605 (7.48%)	1	1
Function unknown	1685 of 32382 (5.20%)	49 of 1605 (3.05%)	1	1
RNA processing and modification	1384 of 32382 (4.27%)	44 of 1605 (2.74%)	1	1

A total of 460 wound-responsive DEGs and 881 defoliation-responsive DEGs were classified into the KEGG pathway. For wounding, the significantly enriched KEGG pathways included valine, leucine, and isoleucine degradation; linoleic acid metabolism; ascorbate and aldarate metabolism; starch and sucrose metabolism; fatty acid elongation; arginine and proline metabolism; and alpha-linolenic acid metabolism (Table 3). For defoliation, starch and sucrose metabolism; ribosome; alpha-linolenic acid metabolism; phenylpropanoid biosynthesis; flavonoid biosynthesis; stilbenoid, diarylheptanoid and gingerol biosynthesis; linoleic acid metabolism; and phenylalanine metabolism were over-represented in the KEGG pathway classification (Table 4).

**Table 3:** KEGG pathways that are enriched in the wounding-responsive DEGs compared with the reference transcriptome.

KEGG pathway	DEGs number	Background number	*P*-Value	Corrected *P*-Value
Valine, leucine and isoleucine degradation	19	71	5.28E-07	3.24E-05
Linoleic acid metabolism	10	23	2.08E-06	9.55E-05
Ascorbate and aldarate metabolism	14	50	9.35E-06	0.000344057
Starch and sucrose metabolism	31	188	1.67E-05	0.000512626
Fatty acid elongation	8	22	0.0001044	0.002745301
Arginine and proline metabolism	19	102	0.0001393	0.003203327
alpha-Linolenic acid metabolism	11	46	0.0003908	0.00798909
Lysine degradation	9	41	0.0024838	0.045702656
Lysine biosynthesis	6	21	0.0032349	0.049957314
Plant hormone signal transduction	17	112	0.0032581	0.049957314

**Table 4:** KEGG pathways that are enriched in the defoliation-responsive DEGs compared with the reference transcriptome.

KEGG pathway	DEGs number	Background number	*P*-Value	Corrected *P*-Value
Starch and sucrose metabolism	62	188	1.85E-10	1.06E-08
Ribosome	91	323	1.97E-10	1.06E-08
alpha-Linolenic acid metabolism	24	46	2.77E-09	1.19E-07
Phenylpropanoid biosynthesis	28	62	8.87E-09	3.18E-07
Flavonoid biosynthesis	15	22	1.94E-08	5.96E-07
Stilbenoid, diarylheptanoid and gingerol biosynthesis	9	11	1.34E-06	3.59E-05
Linoleic acid metabolism	13	23	3.97E-06	9.48E-05
Phenylalanine metabolism	22	62	3.96E-05	0.000850667

### Enrichment of KEGG Pathways Common to Wounding and Defoliation

**Figure 3:**
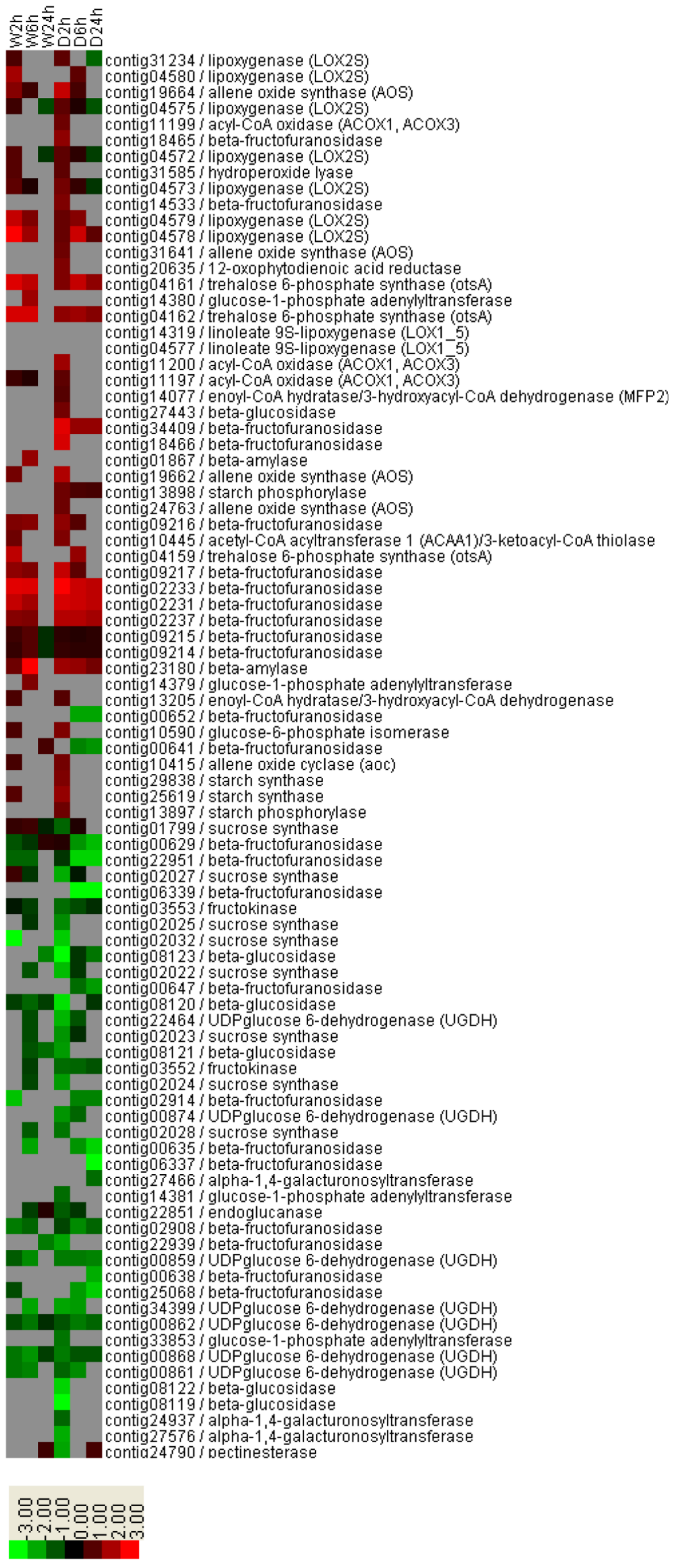
Expression levels of wounding- and defoliation-responsive DEGs with commonly enriched KEGG pathways, including linoleic acid metabolism, alpha-linolenic acid metabolism, and starch and sucrose metabolism. Green to bright red: down-regulation to up-regulation. W, mechanical wounding; D, defoliation.

From the above KEGG pathway enrichment analysis of the DEGs, we observed that linoleic acid metabolism, alpha-linolenic acid metabolism, and starch and sucrose metabolism were enriched KEGG pathways common to both wounding and defoliation. In the alpha-linolenic acid metabolism and linoleic acid metabolism pathways, we identified genes encoding lipoxygenase (LOX), allene oxide synthase (AOS), allene oxide cyclase (AOC), 12-oxophytodienoic acid reductase, acyl-CoA oxidase (ACOX1, ACOX3), acetyl-CoA acyltransferase 1, and enoyl-CoA hydratase/3-hydroxyacyl-CoA dehydrogenase, all enzymes involved in JA biosynthesis. The gene expression levels were significantly increased for wounding and defoliation at 2 h (Figure 3). In starch and sucrose metabolism, genes involved in sucrose metabolism, including genes encoding sucrose synthase, fructokinase, and beta-fructofuranosidase (cell wall invertase), were down-regulated, while genes related to starch biosynthesis and metabolism and trehalose biosynthesis, such as genes encoding starch synthase, starch phosphorylase, beta-amylase, and trehalose 6-phosphate synthase, were up-regulated (Figure 3).

### Defoliation-enriched KEGG Pathways

**Figure 4:**
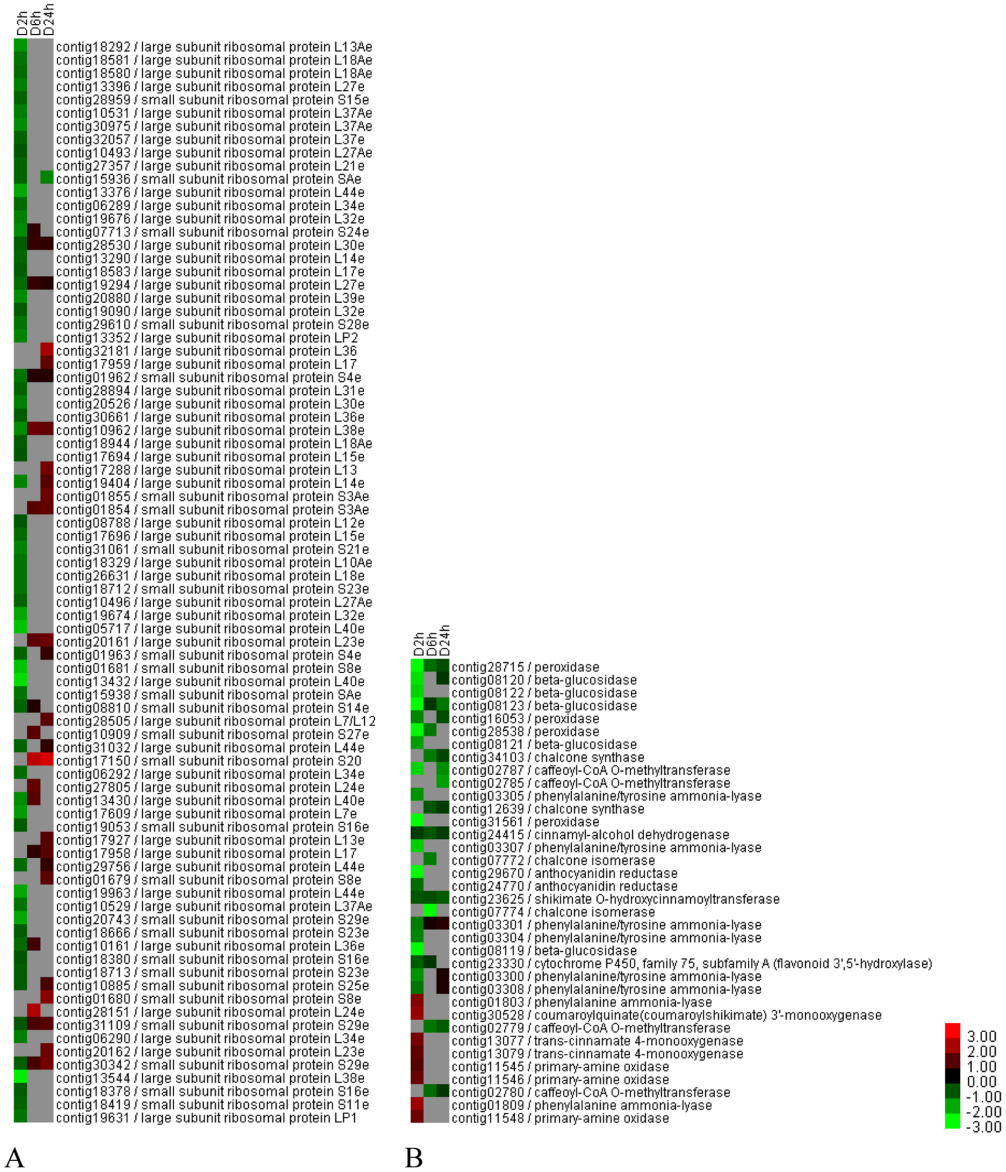
Expression levels of defoliation-responsive DEGs specifically enriched in KEGG pathways. (A) Ribosomal DEGs. (B) DEGs involved in phenylpropanoid biosynthesis; flavonoid biosynthesis; stilbenoid, diarylheptanoid and gingerol biosynthesis; and phenylalanine metabolism. Green to bright red: down-regulation to up-regulation. D, defoliation.

For defoliation, the specifically enriched KEGG pathways were ribosome; phenylpropanoid biosynthesis; flavonoid biosynthesis; stilbenoid, diarylheptanoid and gingerol biosynthesis; and phenylalanine metabolism. Most ribosomal genes were down-regulated after defoliation at 2 h, but some were up-regulated at 24 h (Figure 4A). The expression levels of the genes involved in phenylpropanoid biosynthesis; flavonoid biosynthesis; stilbenoid, diarylheptanoid and gingerol biosynthesis; and phenylalanine metabolism were predominantly decreased (Figure 4B); these down-regulated genes included those encoding cinnamyl-alcohol dehydrogenase, peroxidase, caffeoyl-CoA O-methyltransferase, beta-glucosidase, phenylalanine/tyrosine ammonia-lyase, shikimate O-hydroxycinnamoyltransferase, chalcone synthase, anthocyanidin reductase, and flavonoid 3′,5′-hydroxylase. However, some genes involved in these pathways, including the genes encoding trans-cinnamate 4-monooxygenase, coumaroylquinate (coumaroylshikimate) 3′-monooxygenase, phenylalanine ammonia-lyase, and primary-amine oxidase, were up-regulated (Figure 4B).

### Confirmation of Read-mapped Genes by qRT-PCR

**Figure 5:**
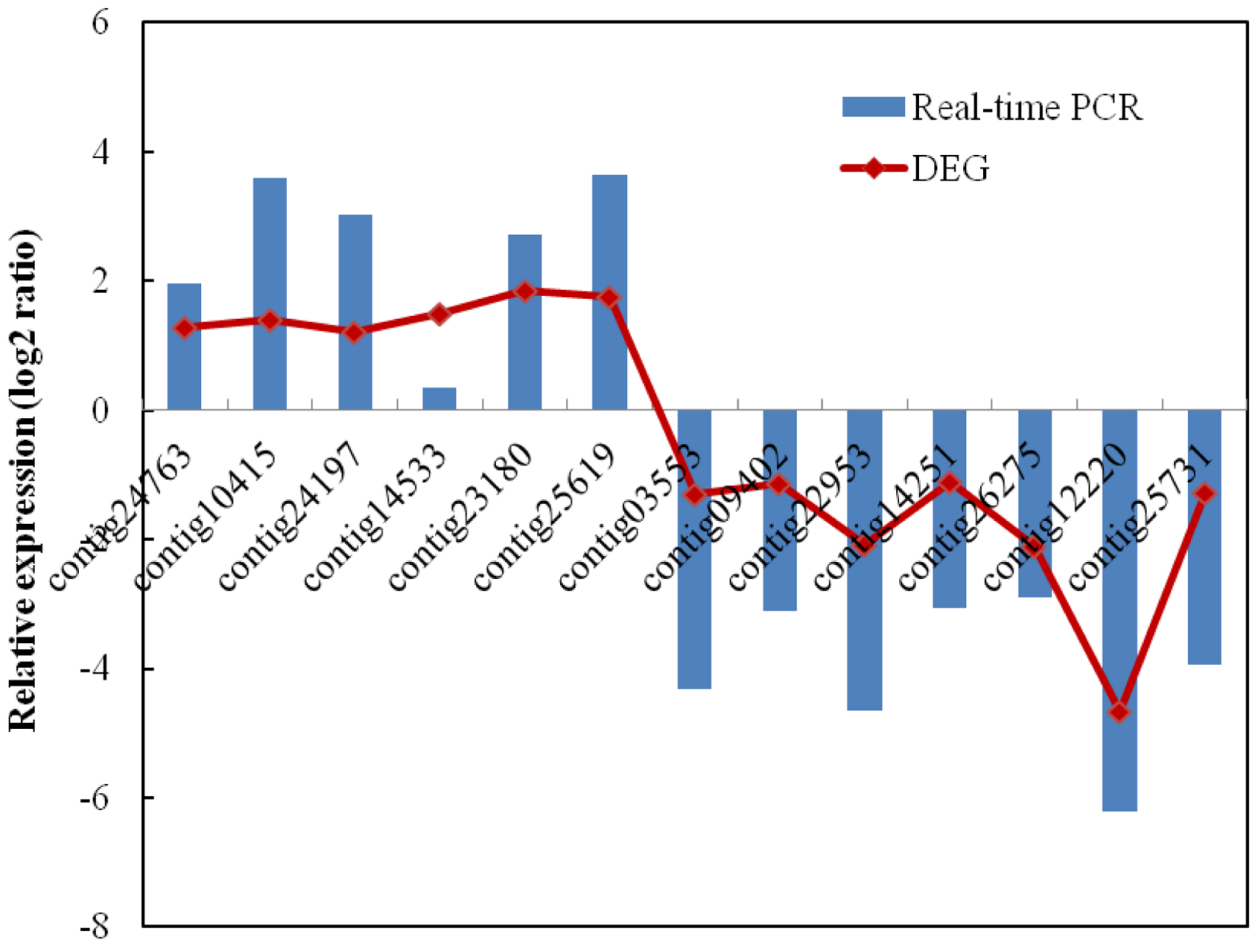
Quantitative PCR validations of read-mapped genes.

To confirm the reliability of the Solexa/Illumina sequencing technology, fifteen genes were selected as described in the Materials and methods for quantitative RT-PCR assays. The expression patterns for thirteen genes were consistent between the qRT-PCR and the DGE analyses (Figure 5). The corresponding primers are listed in Table S1. The inconsistent expression between the qPCR and DGE analyses for the two remaining genes was most likely because DGE was more sensitive in detecting low-abundance transcripts and small changes in gene expression than qRT-PCR.

### Effect of Defoliation on Regrowth of Elongating Leaves

**Figure 6:**
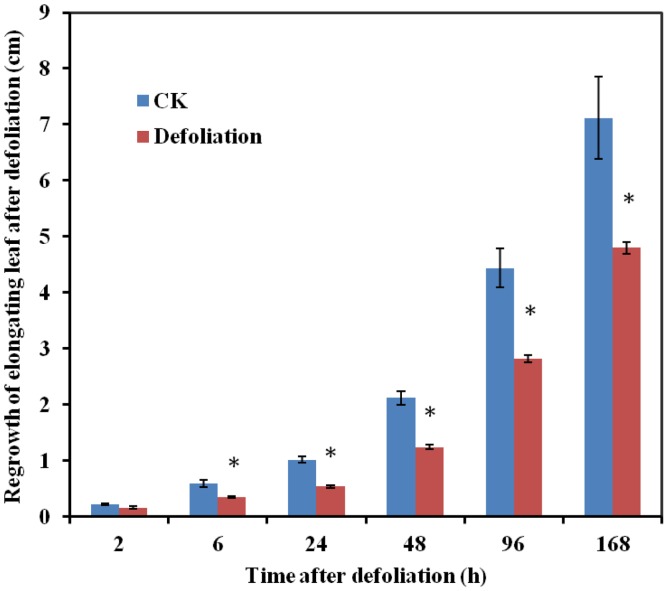
Impact of defoliation on regrowth of elongating leaves above the cutting level. After 8 weeks of growth, sheepgrass plants were defoliated to a stubble height of 5 cm, and regrowth of elongating leaves was measured above the cutting level after defoliation at different time points.

Eight-week-old plants were defoliated to a stubble height of 5 cm. The regrowth of the elongating leaves emerging above the cutting level was measured (Figure 6). The regrowth of elongating leaves was significantly restrained at 6 h after defoliation compared with the growth of the control plants, and the inhibition continued through the following seven days of measurement.

## Discussion

### Distinct Global Gene Transcription Changes for Sheepgrass under Wounding and Defoliation Treatments

In this study, a transcriptome profiling analysis was performed to identify genes that are differentially expressed in wounding- and defoliation-treated plants. A sequencing depth of 4.8 to 6.4 million reads per library was reached (Table S2). The results of the saturation sequencing analyzed in the seven libraries indicated that the sequencing depth was sufficient to cover the transcriptome (Figure S1). We identified 1,836 and 3,238 wounding-responsive and defoliation-responsive DEGs, respectively, which suggested that defoliation led to nearly twice as many DEGs as wounding. Among these DEGs, 954 were uniquely expressed under defoliation and thus represent candidate genes that merit further study. Further analysis indicated that the defoliation-responsive gene number was highest at 2 h, during which the number of down-regulated defoliation-responsive DEGs (1,526) were over four times the number of down-regulated wounding-responsive DEGs (350), suggesting that plants could rapidly respond to the enormous damage of defoliation by regulating global gene expression.

### Wounding and Defoliation Commonly Activate Systemic JA Synthesis

JA is derived from the unsaturated fatty acid α-linolenic acid (18∶3), an abundant octadecanoid in the cellular membranes of higher plants. Lipoxygenase (LOX) is the initial step in JA biosynthesis and catalyzes the oxygenation of α-linolenic acid, leading to the formation of hydroperoxy octadecadienoic acids, which are then converted into 12-oxo phytodienoic acid (12-OPDA) in the plastids via enzymatic reactions catalyzed first by allene oxide synthase (AOS) and subsequently by allene oxide cyclase (AOC). OPDA is then converted to JA by 12-oxo-phytodienoic acid reductase (OPR) followed by three cycles of β-oxidation in the peroxisome [26], [27]. In this study, we observed that the DEGs involved in JA biosynthesis after wounding and defoliation were significantly enriched according to the GO terms and KEGG pathway enrichment analysis (Figure S2, Table 3, 4). The transcription levels of key genes in JA biosynthesis, including genes encoding lipoxygenase (LOX), allene oxide synthase (AOS), allene oxide cyclase (AOC), 12-oxophytodienoic acid reductase (OPR), acyl-CoA oxidase (ACOX1, ACOX3), acetyl-CoA acyltransferase 1, and enoyl-CoA hydratase/3-hydroxyacyl-CoA dehydrogenase (Figure 3), were significantly increased after wounding and defoliation. Our results are consistent with the previous reports, demonstrating that wound-induced responses are triggered by the de novo synthesis of the plant hormone JA when plant tissues are injured by various means, including herbivores, pathogens, and mechanical stress [9], [28]–[29].

### Defoliation Specifically Reduces the Expression Levels of Ribosomal Protein Genes, Cell Division or Cell Expansion-related Genes, and Lignin Biosynthesis Genes and may Negatively Affect Plant Growth

Enrichment analyses of GO terms, KOG, and the KEGG pathway suggested that the ribosome was specifically over-represented after defoliation. The ribosome is an essential ribonucleoprotein complex that is engaged in translation and is indispensable for growth [30]. In this study, many ribosomal protein genes, including the large 60S and small 40S subunits of the cytosolic ribosome, were identified, and their expression levels mainly decreased at 2 h after defoliation (Figure 4A). In both plants and animals, the loss of ribosomal proteins leads to reduced growth, which most likely correlated with reduced ribosome production and lower rates of protein synthesis [31]. For example, in *Arabidopsis thaliana*, several RP loss-of-function mutations that affect cell division or cell expansion and consequently result in a deformed leaf size and shape have been identified, indicating cell- or development-specific roles of RPs during leaf growth [32]. Notably, in this study, the expression levels of numerous genes involved in cell division or cell expansion were down-regulated after defoliation, and these genes include those encoding cyclins, cyclin-dependent kinases, and expansion (Table S4).

Lignin is a phenolic heteropolymer of the secondary cell walls that plays a major role in plant development and defense against pathogens. The monolignols represent the main component of lignin, and their synthesis involves many intermediates and enzymes. In this study, we identified several key genes involved in lignin biosynthesis that were down-regulated, including genes encoding phenylalanine ammonia-lyase (PAL), caffeoyl-CoA O-methyltransferase (CCOMT), shikimate O-hydroxycinnamoyltransferase (HCT), and cinnamyl alcohol dehydrogenase (CAD) (Figure 4B). The down-regulation of these genes has been demonstrated to reduce lignin biosynthesis and cell-wall thickness, alter xylem organization, and retard growth [33]–[36].

Notably, the regrowth of elongating leaves was significantly retarded after defoliation at 6 h through the following 7 days of measurement (Figure 6), suggesting that the above gene expression patterns correlated consistently with the phenotype. Castrillón-Arbeláez et al. demonstrated that although defoliation led to a rapid and transient reduction of non-structural carbohydrates (NSC) in grain amaranth, only a few changes in gene expression and enzyme activity could be associated with the NSC changes in an analysis of 25 genes. They also revealed that the rapid mobilization of foliar starch reserves followed by an efficient recovery of all NSC reserves after defoliation did not alter plant growth or reproductive fitness [17]. Vargas-Ortiz et al. demonstrated that defoliation reduced all NSC levels in the stems and roots of grain amaranth, and this reduction was associated with reduced sucrose synthase and cell wall invertase activity [37]. However, in this study, we observed that the expression levels of genes encoding sucrose synthase and cell wall invertase were reduced under both mechanical wounding and defoliation. Ida et al. also suggested that although defoliation in *Oxytropis sericea* reduced photosynthesis and nectar production, it did not alter photosynthate allocation or fruit or seed production [38]. However, Akiyama and Ågren demonstrated that defoliation in *Arabidopsis thaliana* reduced seed production, and this reduction correlated negatively with the removed leaf area [39]. The above contradictory results suggest that defoliation is a complex process, which includes defoliation timing, intensity, and species tolerance, and may lead to different responses. The highly complex nature of the NSC metabolic flux in plants also indicates that defoliation effects cannot be fully explained at the transcriptional level, and its effects on plant growth and reproductive fitness might be elucidated by systems biology in the future.

## Supporting Information

Figure S1 **Sequencing saturation analysis of the seven libraries.** The number of detected genes was enhanced as the sequencing amount (total read number) increased. W, mechanical wounding; D, defoliation.
(TIF)

Figure S2 **Gene Ontology term enrichment analysis of wounding- and defoliation-responsive DEGs.** (A–C) and (D–F) present the GO terms ‘enrichment status’ and ‘hierarchy’ for wounding and defoliation, respectively; (A and D) the biological process, (B and E) molecular function, and (C and F) cellular component branches. The classification terms and their serial numbers are represented as boxes, and the box includes the GO term, the adjusted p-value (in parentheses), the item number mapping the GO term in the query list and background, and the total number of items in the query list and background. The boxes with significant levels are indicated by color. The color scale from light to dark illustrates the p-value cutoff levels from low to high.
(DOC)

Table S1 **Corresponding primers for qRT-PCR.**
(XLS)

Table S2 **Read number and mapping results for the seven independent libraries.**
(DOC)

Table S3 **DEGs expressed after mechanical wounding.**
(XLS)

Table S4 **DEGs expressed after defoliation.**
(XLS)
