# Supplementary figures and images for: Transcriptome Analysis Reveals Common and Distinct Mechanisms for Sheepgrass (Leymus chinensis) Responses to Defoliation Compared to Mechanical Wounding
Source: PLoS One. 2014 Feb 21;9(2):e89495. doi: 10.1371/journal.pone.0089495 (PMC3931765; doi:10.1371/journal.pone.0089495)

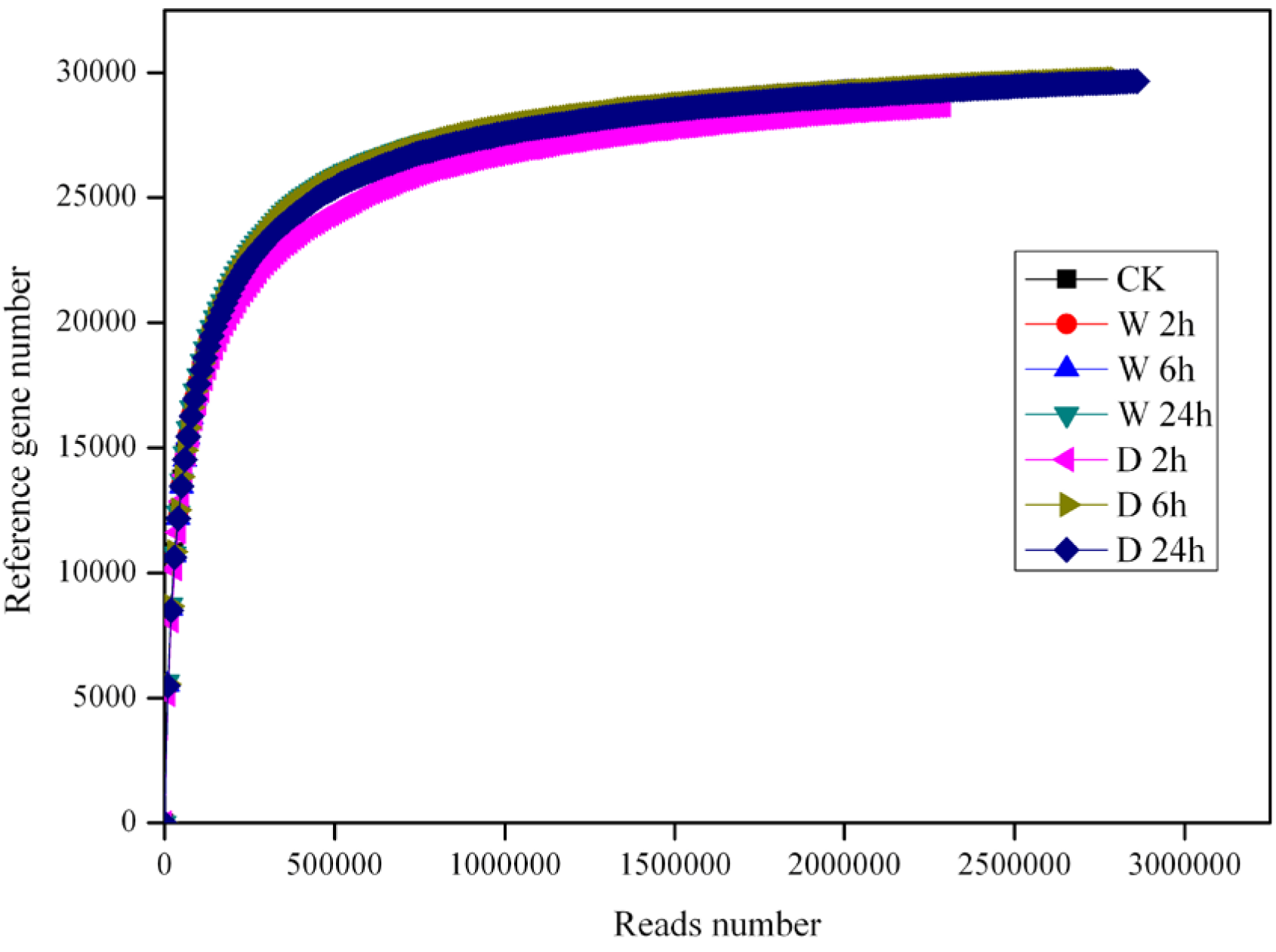

Supplement: Figure S1 — Sequencing saturation analysis of the seven libraries. The number of detected genes was enhanced as the sequencing amount (total read number) increased. W, mechanical wounding; D, defoliation. (TIF) [file pone.0089495.s001.tif]

A


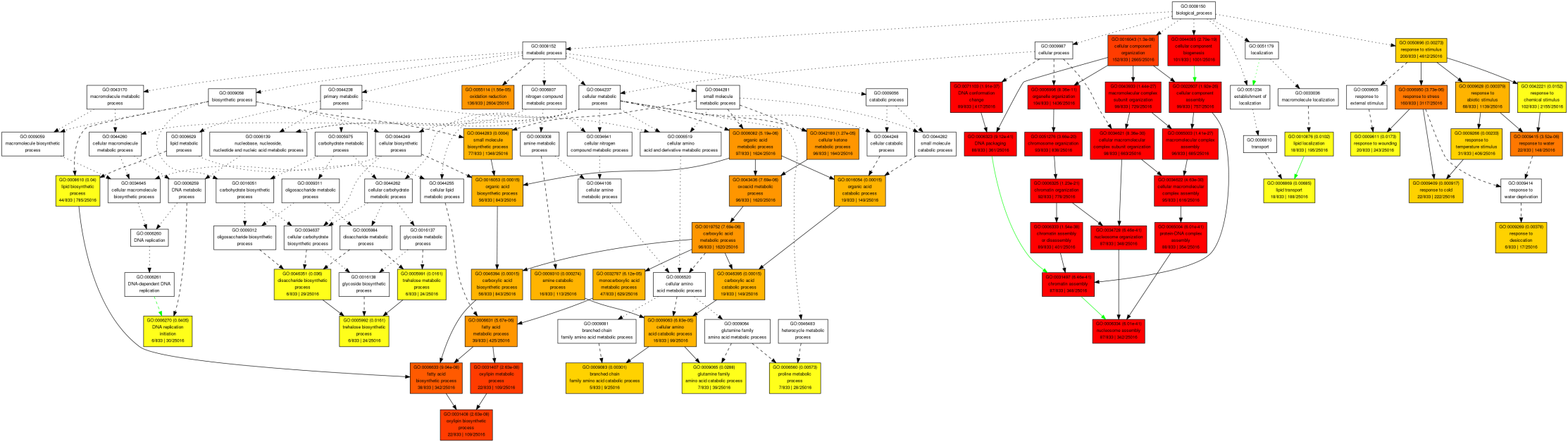


B


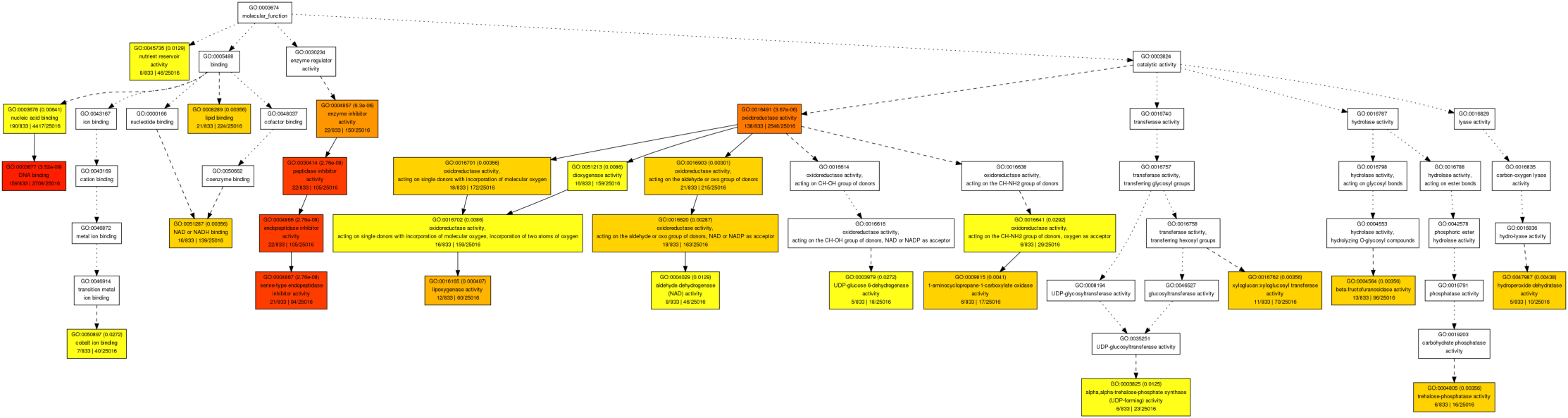


C


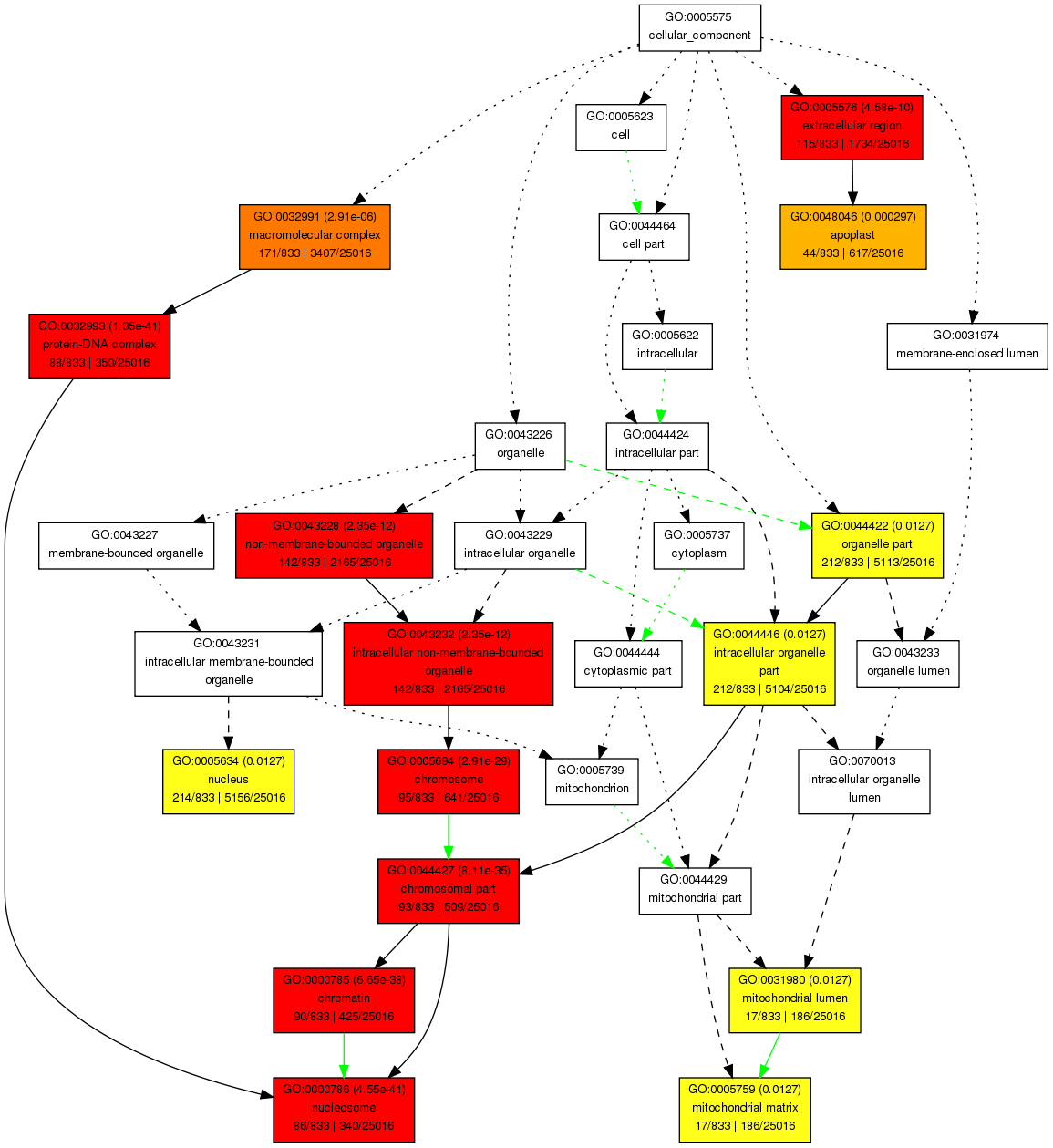


D


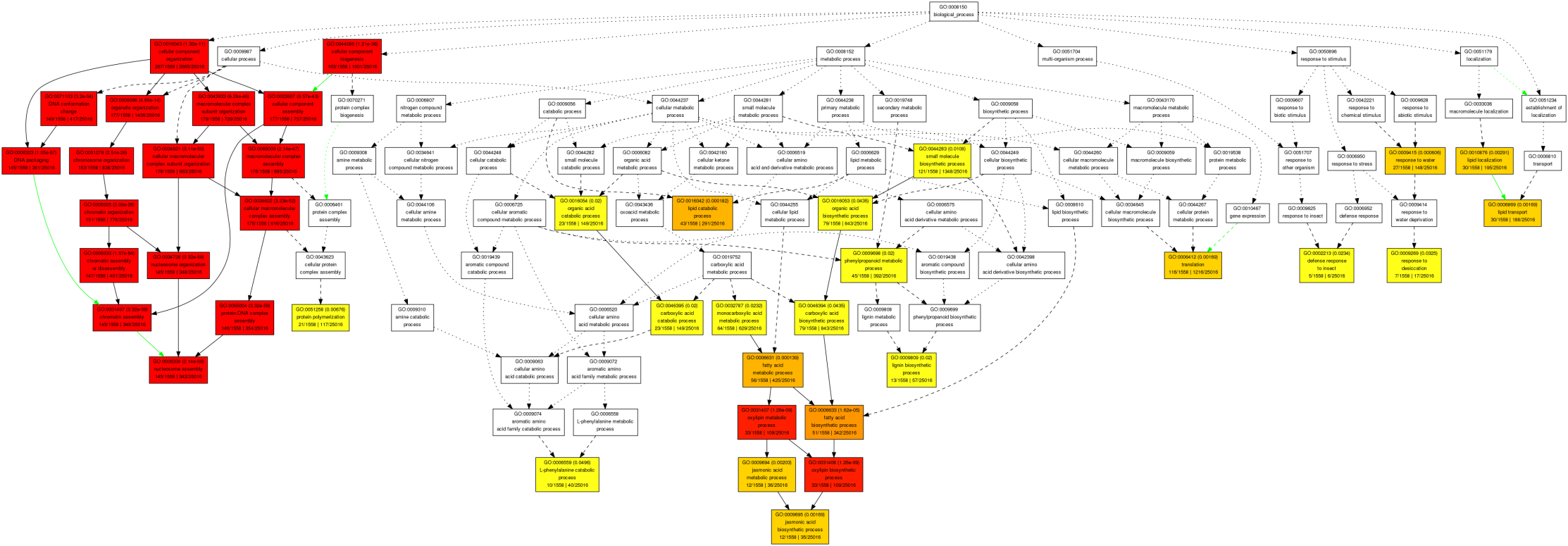


E


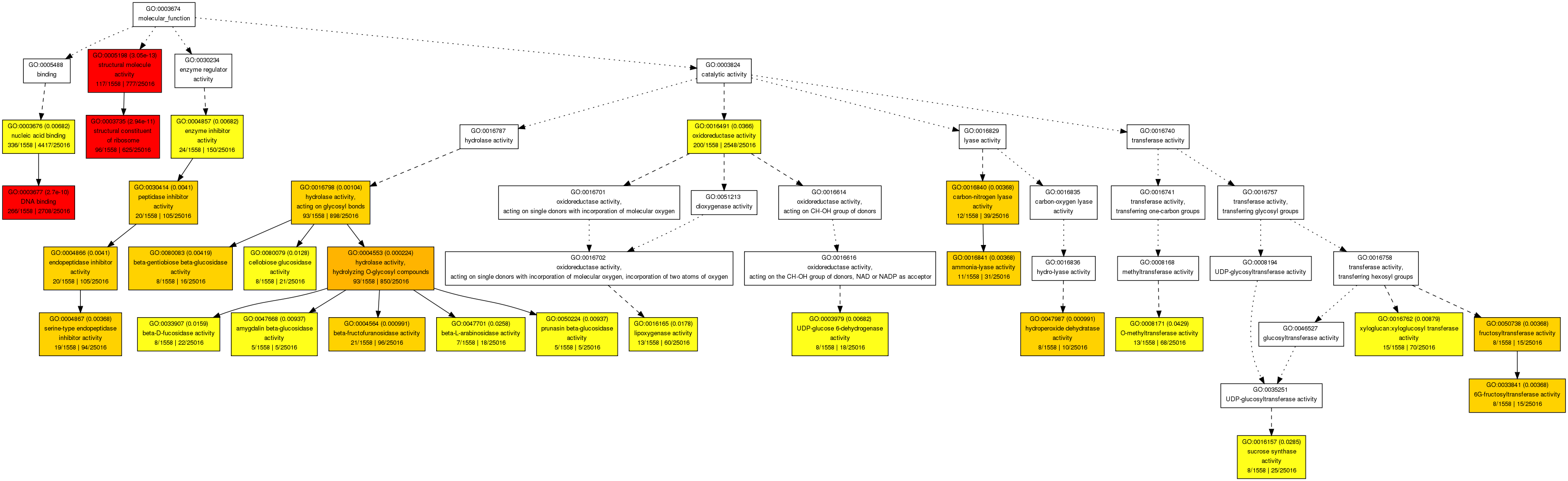


F


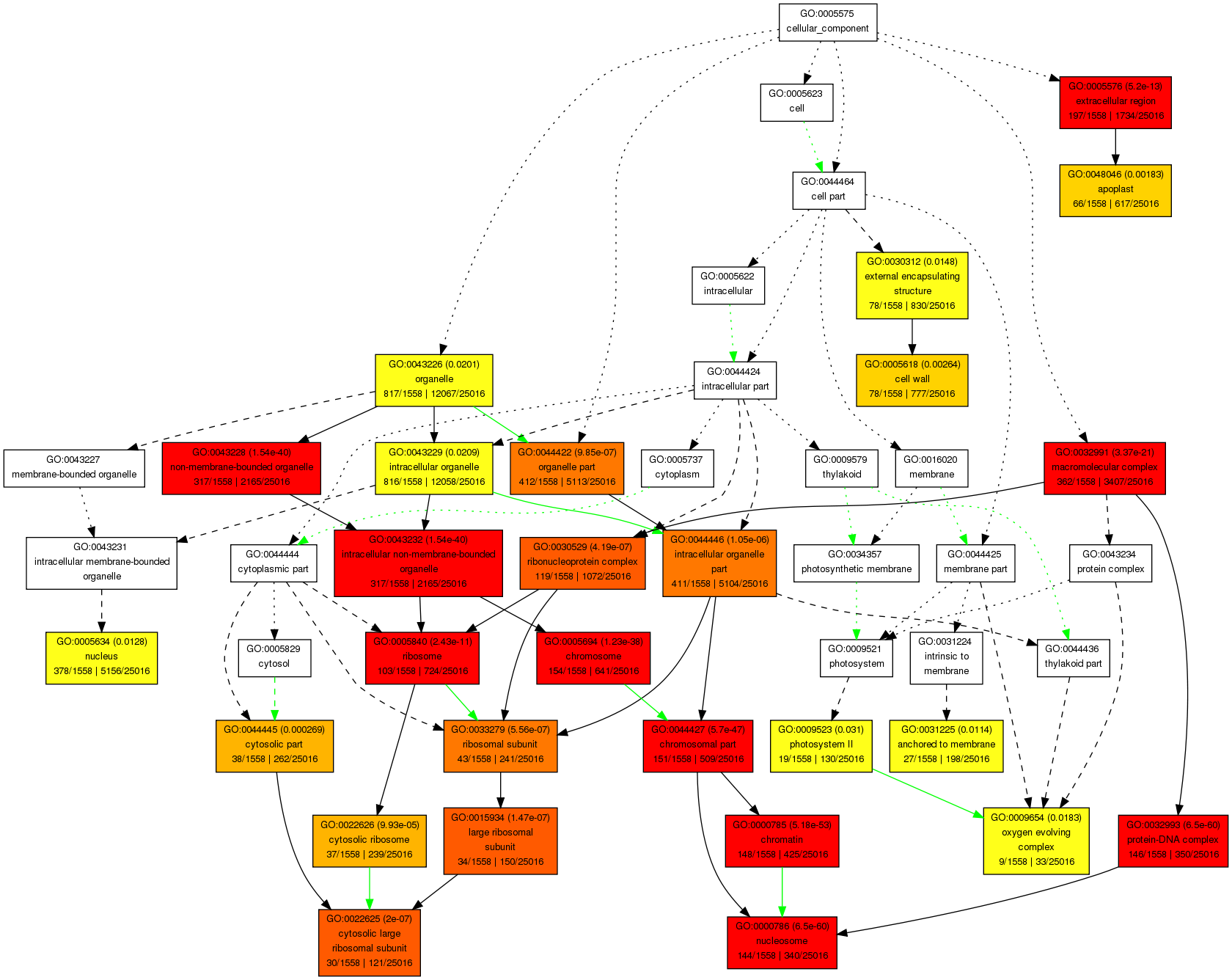

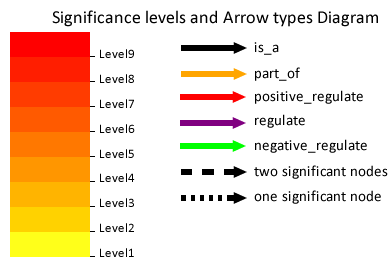

Supplement: Figure S2 — Gene Ontology term enrichment analysis of wounding- and defoliation-responsive DEGs. (A–C) and (D–F) present the GO terms ‘enrichment status’ and ‘hierarchy’ for wounding and defoliation, respectively; (A and D) the biological process, (B and E) molecular function, and (C and F) cellular component branches. The classification terms and their serial numbers are represented as boxes, and the box includes the GO term, the adjusted p-value (in parentheses), the item number mapping the GO term in the query list and background, and the total number of items in the query list and background. The boxes with significant levels are indicated by color. The color scale from light to dark illustrates the p-value cutoff levels from low to high. (DOC) [file pone.0089495.s002.doc]
