# Supplementary material for: Transcriptome Analysis Reveals Common and Distinct Mechanisms for Sheepgrass (Leymus chinensis) Responses to Defoliation Compared to Mechanical Wounding
Source: PLoS One. 2014 Feb 21;9(2):e89495. doi: 10.1371/journal.pone.0089495 (PMC3931765; doi:10.1371/journal.pone.0089495)
Supplement: Table S2 — Read number and mapping results for the seven independent libraries. (DOC) [file pone.0089495.s004.doc]

| Sample name | Control | W 2h | W 6h | W 24h | D 2h | D 6h | D 24h |
| --- | --- | --- | --- | --- | --- | --- | --- |
| Raw reads | 6260824 | 6039667 | 5112646 | 5910902 | 6124061 | 6591077 | 6797308 |
| Clean reads | 5919998 | 5722332 | 4811093 | 5535328 | 5744312 | 6228255 | 6411192 |
| Detected unigenes | 36413 | 35925 | 35113 | 35929 | 34534 | 36828 | 36956 |
